# Advances in Human Brain Dynamics in a Multimodal, Multitask, Multisensory, and Multi-Timescale Framework

**DOI:** 10.3390/brainsci16080824

**Published:** 2026-08-03

**Authors:** Stavros I. Dimitriadis

**Affiliations:** 1 Integrative Neuroimaging Lab, 55133 Thessaloniki, Makedonia, Greece; stidimitriadis@gmail.com; 2 1st Department of Neurology, G.H. ‘AHEPA’, Faculty of Health Sciences, School of Medicine, Aristotle University of Thessaloniki (AUTH), 54124 Thessaloniki, Makedonia, Greece; 3 Neuroinformatics Group, Cardiff University Brain Research Imaging Centre (CUBRIC), School of Psychology, College of Biomedical and Life Sciences, Cardiff University, Maindy Rd., Cardiff CF24 4HQ, Wales, UK; 4 Neuroscience and Mental Health Research Institute, Cardiff University, Maindy Rd., Cardiff CF24 4HQ, Wales, UK; 5 Medical Research Council (MRC) Centre for Neuropsychiatric Genetics and Genomics, Division of Psychological Medicine and Clinical Neurosciences, School of Medicine, Cardiff University, Maindy Rd., Cardiff CF24 4HQ, Wales, UK

The human brain is a dynamic, interconnected system that continuously integrates overlapping streams of information. It operates simultaneously as a multimodal (merging senses), multitasking (juggling functions), multi-timescale, and multisystem processor, using complex spatiotemporal dynamics to construct our conscious experience [1,2]. The human brain generates a rich repertoire of spatiotemporal dynamics during wakefulness, supporting various cognitive functions, task executions, arousal states, and a variety of conscious experiences.

The brain’s architecture perfectly aligns with these capabilities across several core dimensions:

## 1. Multimodal, Multisensational and Multi-Timescale Integration

Our senses do not operate in isolation. The brain integrates visual, auditory, tactile, olfactory, and proprioceptive inputs into a unified perception [3]. Oscillatory neural mechanisms mediate multisensory integration (MI), including power modulations, phase resetting, phase-amplitude coupling, and dynamic functional connectivity. Subsequent studies also indicate the presence of multi-timescale dynamics in intrinsic, ongoing neural activity, as well as during stimulus-driven, bottom-up, and cognitive, top-down neural network processing in the context of MI [4].

- **Multi-timescale Integration:** Multi-timescale operates through three primary mechanisms: (A) fast rhythmic oscillations: In the short term, external sensory rhythms entrain brain activity, producing synchronized firing that binds inputs together. Mechanisms like phase resetting and phase-amplitude coupling instantly link visual, auditory, and somatosensory cortices to prioritize important stimuli. (B) Hierarchical Network Processing: Fast bottom-up sensory inputs are integrated with slower top-down cognitive and attentional signals. This structural hierarchy allows the brain to weigh raw sensory traits against learned predictions or expectations. (C) Long-Term Plasticity: Across the longest timescales, continuous exposure to specific environments structurally rewires neural networks [4].
- **Multisensory Binding:** The brain integrates information from distinct senses (e.g., vision, audition, touch) by coordinating neural dynamics across multiple temporal scales, from milliseconds to years. This dynamic mechanism coordinates rapid oscillatory phase resetting in the milliseconds after a stimulus, up to long-term memory formation spanning a lifetime. Specialized regions (such as the superior temporal sulcus) combine disparate sensory signals, such as matching lip movements to speech, to enhance overall perception [4]. This audiovisual paradigm facilitates sensory binging in three different time-scales linked to different events: A) Fast Timescale (~10 to 50 ms)—Sensory Encoding, B) Medium Timescale (~50 to 150 ms)—Temporal Window of Integration, and C) Slow Timescale (~200 ms+)—Top-Down Binding & Decision.
- **Neural Oscillations:** Different sensory cortices synchronize their firing rhythms (e.g., in the β band) to bind fragmented sensory data into a single conscious experience. In an audiovisual serial prediction task, participants had to acquire stimulus sequences and to monitor whether subsequent probe items complied with the sequence. Researchers reported that multi-timescale neural dynamics underlying multisensory predictions, suggesting that oscillations in multiple frequency ranges (θ and β), as well as coupling within and across frequencies (cross-frequency coupling), may be critical for multisensory sequence processing [5].
- **Cross-Modal Plasticity:** When one sense is impaired, the brain can rewire itself to allocate idle cortical regions to process remaining sensory inputs [6].
- **Top-Down Transfer:** Because multisensory processing integrates meaning and associations, it benefits from automatic top-down transfer of training. This transfer allows the brain to generalize skills more effectively than traditional, single-sense training methods [7,8].
- **Individual Differences:** The nature of multisensory integration and plasticity varies across individuals. Factors such as an individual’s distinct sensory experiences, personality traits, and neurodevelopmental profiles affect how they integrate different sensory inputs [9].

## 2. The Multitask and Multiple Demand (MD) System

While humans struggle with conscious, simultaneous high-level tasks, the brain’s underlying networks naturally handle multiple background operations simultaneously [10].

- **The Multiple Demand (MD) Network:** This core set of distributed regions (including the lateral prefrontal cortex and intraparietal sulcus) flexibly reallocates cognitive resources to meet moment-by-moment task demands [10,11].
- **Shared Representations:** Rather than building from scratch for each task, the brain uses overlapping, shared neural representations to generalize learning and transfer knowledge across environments [10,11].
- **Dynamic Routing:** Large-scale networks (such as the frontoparietal and default mode networks) continuously shift in and out of synchrony to support shifting attention and motor control [12,13,14].

## 3. Multisystem Hierarchy

The brain coordinates activity across multiple distinct physiological and anatomical systems to function seamlessly.

- **The Perceptual-Action Loop:** It closes the loop instantly, turning multisensory perceptions into motor commands within milliseconds using complex feedback loops [15,16]. Κey mechanisms of the **Perceptual-Action Loop are** as follows:

**A. Sensory Encoding:** Sensory organs capture raw environmental data. The brain immediately filters out expected, self-generated changes to isolate actionable information [17].

**B. Motor Commands:** Using pathways such as the cortical perception-action network, multisensory data is translated into precise motor commands [18].

**C. Rapid Feedback:** Proprioceptive and sensory feedback is routed back to early processing areas, enabling instant, reflex-like corrections and stabilizing physical control [18].

- **Whole-Brain Dynamics:** Function relies heavily on the interplay among the brainstem (arousal), the limbic system (emotion), and the cerebral cortex (higher-order reasoning) [19,20].
- **Hierarchical State Trajectories:** Brain activity naturally forms low-dimensional, fluid patterns, or “manifolds,” that shift as internal states or environmental requirements change [21].

Neural dynamics continuously reconfigure to support cognitive function. During experimental tasks, specific functional networks synchronize to meet task demands [22]. Across different arousal states and levels of wakefulness, neuromodulatory systems alter global brain connectivity, shifting dynamics among focused attention, mind-wandering, and sleep [23,24,25,26].

The brain’s architecture perfectly aligns with these capabilities across several core dimensions that support the reconfiguration. These are:

- **Task Execution:** Brain networks exhibit flexible functional connectivity. The brain rapidly transitions between integrated states (sharing information across widespread regions) and segregated states (processing locally) to optimize performance [3,27].
- **Multisensory advantage in learning:** Multisensory perception and plasticity reveals that human perception is not a series of independent channels but a unified stream in which the senses continuously interact to interpret reality. The brain flexibly rewires itself—a process known as neuroplasticity—to integrate cross-modal information, compensate for sensory loss, and dramatically accelerate learning [28].
- **Arousal States:** Neuromodulators such as acetylcholine, noradrenaline, and dopamine modulate neuronal excitability and the signal-to-noise ratio. High arousal sharpens focus, while low arousal leads to broader, less structured neural firing [29,30].
- **Consciousness:** Transitions between wakefulness, sleep, and anesthesia involve large-scale shifts in complexity. Conscious states are characterized by high-dimensional, complex neural dynamics that enable rich information integration, whereas unconscious states exhibit reduced, highly stereotyped, or fragmented activity [31,32].

The organization of the brain encompasses molecular-level processes within neurons as well as large-scale neural networks. Understanding this multiscale structure is crucial for elucidating brain functions and addressing neurological disorders [33]. Multiscale brain modeling has become a transformative approach that integrates computational models, advanced imaging techniques, and large-scale data to connect these organizational levels [2]. Relevant brain scales span from the molecular organization of DNA and proteins to cells, gene expressions (transcriptomics), circuits, large-scale networks, and human social interactions, as they all interface with brain structure.

Advanced computing architectures (such as MAMBAxBrain frameworks) and artificial neural networks are now drawing inspiration from the brain’s holistic, multiscale organization to improve efficiency and generalize across multiple cognitive tasks [34].

### 3.1. The Complexity of Sensory Integration

**Sensory integration** refers to the brain’s capacity to combine input from different sensory modalities, resulting in a unified perception of the environment. Sensory systems function interdependently, with the brain integrating signals from multiple sources to generate a holistic and conscious experience [35]. This process relies on neural oscillations (brain rhythms) to coordinate communication across distinct brain regions. Rhythms such as α, β, and γ waves allow these areas to synchronize, selectively routing information and binding sensory features [36,37]. This integration engages several brain regions, each specialized for distinct aspects of sensory processing, yet collectively contributing to coherent perception [38]. Neural mechanisms that integrate and segregate multisensory information are essential for complex scene analysis and for addressing the multisensory correspondence problem [39,40]. A recent study revealed how the human brain solves the “multisensory correspondence problem”—deciding whether auditory and visual signals belong to the same source. The researchers used a time-resolved encoding model based on the Multisensory Correlation Detector (MCD). Unlike older, static models of cue combination (like Bayesian optimal integration), the time-resolved MCD reveals that multisensory perception relies on rapid, dynamic, single-trial computations of signal alignment over time [3]. The brain’s ability to synthesize sensory data is fundamental for environmental interaction, decision-making, and effective response.

### 3.2. Neural Mechanisms of Sensory Integration

Sensory integration depends on complex networks of brain regions, particularly within the **parietal, temporal, and occipital cortices** [5]. These regions are essential for coordinating and integrating sensory information across multiple modalities. The following examples illustrate these processes:

- **Multisensory Neurons**: Certain neurons are capable of processing information from multiple sensory modalities. These neurons are located in regions such as the **superior temporal sulcus (STS)**, where visual and auditory information is integrated. This neural activity enables the brain to associate visual stimuli, such as lip movements, with auditory inputs, such as speech sounds, to generate coherent perception [41].
- **Cross-modal Integration**: The **posterior parietal cortex** is a critical region for integrating sensory information. It facilitates the integration of inputs from different modalities, such as vision and touch, enabling accurate environmental perception and effective navigation [42]. This integration also underpins **spatial awareness**, which is essential for complex tasks such as driving or walking.
- **Attention and Modulation**: Sensory integration is an active process in which attention modulates the prioritization of sensory information. For example, during a conversation in a noisy environment, the brain suppresses irrelevant sensory inputs, such as background noise, to enhance speech clarity. The **frontal cortex** and **thalamus** are involved in regulating attention, ensuring that sensory integration aligns with behavioral goals and environmental demands [43].

This **research topic** presents eight contributions addressing various aspects of human brain dynamics, neuroimaging modalities, and target sensory systems.

Parameshwaran and Thiagarajan (2023) proposed a novel approach to characterizing amplitude variability in the EEG signal, termed “High Variability Periods” (HVPs). HVPs define these periods as segments in which the standard deviation within a moving window consistently exceeds the quartile cutoff. HVPs have been evaluated against alternative single-channel variability estimators and across diverse experimental conditions. HVP metrics enhanced discrimination among distinct brain states, enabled rapid estimation, and provided a complementary perspective on signal variability, suggesting potential clinical utility for swift brain state identification (Contribution 1).

Advancements in neurotechnology have positioned **brain–computer interfaces (BCIs)** as promising tools for investigating sensory integration. BCIs facilitate direct communication between the brain and external devices, thereby enabling the enhancement or modification of sensory input. For instance, BCIs may bypass damaged sensory pathways, permitting individuals with sensory impairments to perceive their environment through alternative modalities. Recent progress in invasive BCIs, propelled by advances in electrode materials, miniaturized, energy-efficient electronics, and neural signal decoding technologies, has attracted considerable scholarly interest. Zhao et al. (2023) provided a review outlining the fundamental principles of neuronal signal decoding and encoding that underpin BCI-mediated information transfer. The review focused on research studies that used neural signals to control external devices and modulate δ brain activity. In the context of brain activity modulation, the authors discussed two principal techniques: electrical stimulation of cortical tissues and electrical stimulation of deep-brain tissues. The review concludes by addressing ethical considerations associated with the clinical implementation of this emerging technology (Contribution 2).

Recent advancements in visual encoding models for functional magnetic resonance imaging have utilized deep neural networks, particularly convolutional neural networks (CNNs) such as VGG16. Standard CNNs typically employ small kernel sizes, such as 3 × 3, for feature extraction. Biological evidence indicates that neuronal population receptive fields in high-level visual encoding regions are three to four times larger than those in low-level regions. Therefore, CNNs with larger receptive fields align more closely with biological observations. The RepLKNet model addresses this by expanding the convolution kernel size to achieve a larger-scale receptive field. This study introduced a hybrid model that integrated RepLKNet and VGG, leveraging their respective receptive fields to capture more comprehensive image features. Experimental results indicated that the hybrid model surpasses conventional convolutional models in encoding performance across multiple regions of the visual cortex. These results suggested that incorporating larger-scale receptive fields into visual encoding models improves the effectiveness of convolutional networks for visual representation (Contribution 3).

Agbangla et al. (2022) examined changes in behavioral performance, subjectively perceived difficulty, and prefrontal cortex hemodynamic activity as a function of cognitive load during two executive function tasks. The authors also assessed relationships among behavioral, subjective, and neuroimaging measures. Nineteen right-handed young adults (aged 18–22 years) underwent continuous-wave functional near-infrared spectroscopy while performing n-back and random number generation tasks under three cognitive load conditions. Findings indicated that cognitive load affects executive performance and perceived difficulty. This study concluded that functional near-infrared spectroscopy can clarify the prefrontal cortex’s role in executive tasks involving inhibition and working memory updating (Contribution 4).

Olfactory hedonic evaluation represents the primary dimension of olfactory perception and is central to the sense of smell. This process involves complex interactions among brain regions associated with sensory, affective, and reward processing. Specifically, the communication and interaction among brain areas during the hedonic evaluation of olfactory stimuli with varying levels of pleasantness, as well as the underlying oscillatory mechanisms, remain poorly understood. In this study, changes in functional interactions among brain areas in response to odor stimuli were investigated using electroencephalography (EEG), the phase synchronization index for brain connectivity analysis, and graph theory. The findings indicate that odor stimuli with different hedonic values evoked significantly distinct interaction patterns among brain regions within the olfactory cortex, anterior cingulate cortex, and orbitofrontal cortex. Additionally, significant hemispheric laterality effects were observed in the prefrontal and anterior cingulate cortices, particularly in the β (13–30 Hz) and γ (30–40 Hz) frequency bands (Contribution 5).

Dimitriadis (2022) investigated, for the first time, virtual source connectivity within and between brain frequencies across the whole brain using the AAL atlas in a lifespan cohort. Source activity was extracted from resting-state magnetoencephalography data obtained from 103 subjects aged 18 to 60 years. This cohort was collected with an ELEKTA MEG system. The directionality of information flow was assessed using regional time courses analyzed with delay symbolic transfer entropy and phase entropy. This approach generated a dynamic source connectivity profile that distinguished the direction, strength, and time delay of underlying causal interactions, providing independent time delays for cross-frequency amplitude-to-amplitude and phase-to-phase coupling. Calculation of the dominant intrinsic coupling mode (DoCM) allowed for independent estimation of the probability distributions of DoCM for both phase and amplitude. The DoCM between intra- and cross-frequency couplings (CFC) was presented here for the first time, separately for amplitude and phase. In both domains, {δ, θ, α_1_} frequencies were the primary contributors to DoCM. He introduced a novel brain age index (BAI), defined as the ratio of the probability distributions of inter- and intra-frequency couplings. This ratio demonstrated a universal age trajectory, characterized by a rapid increase following adolescence, a peak in adulthood, and a gradual decline thereafter. This consistent pattern was observed in the BAI across all frequencies examined and in both the amplitude and phase domains. Such a universal age dependence, evaluated using data from a second external dataset recorded with a CTF MEG system, has not been reported previously (Contribution 6).

Visual encoding models utilizing deep neural networks (DNNs) demonstrate strong predictive performance for brain activity in low-level visual areas. However, limitations in neural data hinder the effective fitting of DNN-based models to high-level visual areas, leading to suboptimal encoding performance. The ventral stream indicates that higher visual areas receive information from lower visual areas, a hierarchical pattern not fully captured by current encoding models. Xiao et al. (2022) introduced a novel visual encoding model framework that leveraged the hierarchical organization of representations in the ventral stream to enhance performance in high-level visual areas. The framework comprised two categories of hierarchical encoding models, developed from voxel and feature perspectives to implement hierarchical representations. From a voxel perspective, an encoding model was first constructed for the low-level visual area (V1 or V2), and the predicted voxel space was then extracted. This extracted voxel space was then used to predict the voxel space of the high-level visual area (V4 or Lateral Occipital (LO)) by constructing a voxel-to-voxel model. Experimental results indicated that both categories of hierarchical encoding models substantially improved encoding performance in V4 and LO, providing higher prediction accuracy than other models (Contribution 7).

Hippocampal-sparing radiotherapy (HSR) represents a promising strategy to reduce cognitive side effects associated with cranial radiotherapy. Microstructural brain changes following irradiation have been demonstrated using Diffusion Tensor Imaging (DTI). Dinkel et al. (2022) investigated the effects of radiation on white matter and hippocampal microstructure using DTI and evaluated the potential mitigation of these effects through HSR. The study enrolled thirty-five patients with tumors in a prospective randomized controlled trial who received either conventional radiotherapy or HSR and underwent DTI before treatment and at 6, 12, 18, 24, and 30 (±3) months post-radiotherapy. Hippocampal FA increased at 6 and 12 months, primarily due to increases in Axial and Mean Diffusivity, whereas Radial Diffusivity did not increase until 30 months post-radiotherapy. Mean radiation dose showed a positive correlation with hippocampal Fractional Anisotropy. These findings suggested complex pathophysiological changes in cerebral microstructures following radiation that conventional DTI models did not fully capture. After 6 months, hippocampal microstructure differed between patients receiving HSR and those undergoing conventional cranial radiotherapy, with the HSR subgroup exhibiting higher Axial Diffusivity (Contribution 8).
